# Disparities in Postoperative Endocrine Outcomes After Endoscopic-Assisted Transsphenoidal Pituitary Adenoma Resection

**DOI:** 10.7759/cureus.31934

**Published:** 2022-11-27

**Authors:** Chhitij Tiwari, Eugene Maung, Aaron Gelinne, Nathan Quig, Brian Thorp, Adam Zanation, Matthew Ewend, Deanna Sasaki-Adams, Carolyn Quinsey

**Affiliations:** 1 Department of Neurosurgery, University of North Carolina at Chapel Hill, Chapel Hill, USA; 2 Department of Otolaryngology - Head and Neck Surgery, University of North Carolina at Chapel Hill, Chapel Hill, USA; 3 Department of Otolaryngology - Head and Neck Surgery, Carolina Ear Nose & Throat, Hickory, USA

**Keywords:** diabetes insipidus, endoscopic sinus surgery, pituitary adenoma, socioeconomic disparities, skull base

## Abstract

Objectives

Socioeconomic factors can influence morbidity in patients with pituitary adenoma. This study aims to identify associations between socioeconomic factors and postoperative outcomes in patients with pituitary adenomas.

Methods

A retrospective medical chart review was conducted on adult patients who underwent resection of purely sellar nonfunctional and functional pituitary adenomas between May 1, 2014, and May 31, 2020, at the University of North Carolina Medical Center. The main outcome measures included the incidence of postoperative diabetes insipidus (PDI), postoperative hyponatremia (PHN), and postoperative hypopituitarism (PHP). Outcome measures were analyzed using univariate and multivariate analyses against preoperative tumor volume as well as socioeconomic and demographic factors (self-identified race/ethnicity, age, gender, address assessed by the Area Deprivation Index (ADI), and insurance status).

Results

On univariate analysis, patients of Hispanic race/ethnicity and patients living in more disadvantaged neighborhoods had an increased incidence of postoperative diabetes insipidus. Patients who experienced PDI were significantly younger on average in both univariate and multivariate analyses. When analyzed further, patients of Hispanic race/ethnicity were significantly younger and more likely to be uninsured compared to their respective racial/ethnic counterparts. No significant correlations were found for PHN or PHP.

Conclusions

Patients of Hispanic race/ethnicity and patients living in more disadvantaged neighborhoods were more likely to experience PDI. This finding, when combined with findings regarding age and insurance status, suggests complex disparities in medical care that are confirmed or corroborated by prior literature. These results may enhance clinicians’ management of patients from disadvantaged socioeconomic backgrounds through increased awareness of disparities and the provision of resources for assistance.

## Introduction

Disparities in healthcare access in the United States are well documented in the medical literature [1,2]. These disparities are often worst for racial and ethnic minorities, uninsured patients or patients on Medicaid, and patients of lower socioeconomic status, resulting in greater mortality rates and deficiencies in disease screening [3-5]. Due to persistent structural racism, minority patients are limited in their ability to access high-quality healthcare, their ability to be equally and fairly treated in a medical setting compared to non-minority patients, and their potential opportunities for healthy lifestyle provision [6]. Regional socioeconomic disparities, as defined by the Area Deprivation Index (ADI), are similarly correlated with increased mortality and screening deficits from oncologic diseases [7,8].

These healthcare disparities exist in neurosurgery as well. Patients living in regions with higher socioeconomic disparities, as measured by a higher ADI, have been shown to have higher mortality rates from central nervous system (CNS) tumors and decreased access to postoperative care following tumor resection [9,10]. Racial/ethnic minorities, uninsured patients, and patients with public insurance have similarly been shown to experience greater disease burden, higher morbidity following neurosurgical treatment, and increased incidence of postoperative complications and death [11-13]. These demographic and socioeconomic patterns also exist in the incidence of symptomatic pituitary adenomas. For example, one study noted that a disproportionate percentage of pituitary adenoma patients were likely to be African American; patients with pituitary adenomas were also likely to be older, and male patients were likely to have larger tumors at initial presentation [14]. Female patients were more likely to present earlier in life, while male patients were more likely to present later [14].

These patterns also hold true for patient morbidity prior to pituitary adenoma resection, as well as the likelihood of postoperative complications following endoscopic transsphenoidal resection [15-18]. Public insurance status, for example, has been associated with a larger average tumor diameter and a longer postoperative length of stay in the hospital [15,16]. Lower education has been noted as a risk factor for long-term work disability, as well as an increased rate of health-related absence from work, following pituitary adenoma resection [17]. Lower mean household income, lack of insurance, and lack of a primary care provider have been associated with an increased risk of pituitary apoplexy [18].

Few studies, however, have specifically looked at socioeconomic disparities in postoperative endocrinopathies. These endocrinopathies include postoperative diabetes insipidus (PDI), postoperative hypopituitarism (PHP), and postoperative hyponatremia (PHN); they often require hormonal replacement therapy and/or fluid restrictions for management and add significant medical burden to patients’ lives [19,20]. While a few studies have looked at demographic patterns in the incidence of PDI, they demonstrate findings that directly contradict each other, yielding little overall consensus. Ajlan et al., for example, found that patients under 50 years of age were significantly more likely to experience PDI [21]. However, a similar study conducted around the same time by Nayak et al. found no demographic correlates with PDI [22].

Our study is the first of its kind to specifically look at postoperative endocrinopathies of transsphenoidal pituitary adenoma resection and determine socioeconomic and demographic correlates. We specifically examine patients who have undergone resection through endoscopic surgery, which has shown significant promise as a less invasive technique in a broad array of head and neck surgeries including cochlear implantation [23]. Our aim was to review our institutional records for multiple endocrine complications, including PDI, PHN, and PHP, and determine whether any demographic or socioeconomic markers correlated significantly with an increased likelihood of experiencing any of these complications.

This work was presented as a meeting abstract and poster at the virtual North American Skull Base Society conference on February 13, 2021.

## Materials and methods

Our institutional review board approved this study prior to data collection and granted a waiver for obtaining informed consent (approval number: 20-0994). We conducted a retrospective chart review of 216 patients over 18 years of age who underwent transsphenoidal surgery for the removal of a functional or nonfunctional pituitary adenoma between May 2014 and May 2020 at our institution. To be included in our sample, patients had to be formally diagnosed with a pituitary adenoma in their final pathological specimen notes.

Factors of interest

Socioeconomic factors and demographic variables were collected from the patients’ medical records and included insurance status, age at the time of surgery, address at the time of surgery, and sex at birth (male/female).

Insurance status was stratified into Private, Public, or None. Patients on Medicare, Medicaid, or State Health Plan insurance as their primary coverage were coded as Public, patients with other commercial insurance policies were coded as Private, and patients with no insurance policy at all were coded as None.

The racial cohorts considered in the study were Whites and African Americans, while the ethnic cohorts considered were Non-Hispanic and Hispanic. These cohorts were based on a patient’s self-identified racial and ethnic identity. Based on the combined racial and ethnic fields displayed on patient charts, racial and ethnic cohorts were combined into Non-Hispanic White, Non-Hispanic African American, and Hispanic for purposes of data analysis. Patients whose racial/ethnic status was Unknown, Unreported, Other, or Mixed were to be excluded from the study.

Addresses were evaluated using Kind’s Neighborhood Atlas to create an Area Deprivation Index for each patient and then sorted according to increasing (i.e., worsening) ADI percentile for analysis [24].

Preoperative tumor volume was calculated from dimensions obtained from formal radiology interpretations of preoperative MRI scans. To be included in our study, both imaging and interpretation had to have been conducted within six months prior to tumor resection. Tumor volume was approximated using the traditional calculation: ½ (length × width × height) [25,26].

Outcomes of interest

We measured the incidence of PDI, PHN, and PHP to assess endocrine complications following surgery. For a patient to be noted as having PDI, they had to have received a prescription for desmopressin at the time of discharge. PHN was defined as a persistent sodium level of less than 130 mmol/L, with or without symptoms, for at least two consecutive inpatient days requiring fluid restriction.

Each patient’s hormonal function was assessed through a review of preoperative and postoperative endocrinology reports. In particular, formal diagnoses of hormonal abnormalities, including hypothyroidism, hyperthyroidism, hypoadrenalism, Cushing’s disease, acromegaly, and hyperprolactinemia, were noted. Endocrinology reports at three months and one year postoperatively were evaluated for changes in hormonal function after surgery. Hormonal function was noted as “Restored” if there was an overall recovery from preoperative endocrinopathy by hormonal axis by one year, as assessed by formal endocrinology evaluation. Function was noted as “Unchanged” if formal diagnoses of hormonal abnormalities remained the same by one year and if no new abnormalities were noted. If any new hormonal dysfunction was formally diagnosed after surgery and persisted for one year, this was noted as postoperative hypopituitarism (PHP) [27,28].

Statistical analysis

All statistical analyses were performed using R (R Core Team, Vienna, Austria). ADI, sex, race/ethnicity, age at the time of operation, preoperative tumor volume, and insurance status were used as the primary independent variables, and PDI, PHN, and PHP were used as the primary dependent variables. Univariate and multivariate analyses were conducted for each primary dependent against all primary independent variables. Significance was determined at a p-value of less than 0.05. Associations are reported as odds ratios (OR) with corresponding 95% confidence interval (CI). Subsequent exploratory univariate and multivariate analyses were conducted using race/ethnicity as a primary dependent variable against all other primary independent variables.

## Results

Study population

After querying patients from our database through Current Procedural Terminology (CPT) codes, a total of 300 patients with purely sellar tumors were screened in our initial search of pituitary adenoma patients. We excluded 84 patients from this query due to a non-transsphenoidal procedure (N = 1) or intraoperative pathological specimen report negative for pituitary adenoma (N = 83), resulting in a pool of 216 patients for analysis. Out of these, six patients were excluded from analysis because their preoperative radiology reports lacked formally assessed tumor dimensions (N = 3) or because an ADI could not be calculated for their address (N = 3), resulting in a final pool of 210 patients.

Patient characteristics

Detailed information on patient characteristics is shown in Table 1. The average age of our patient pool is 52.3 years. Our patient pool is 50% male, 56% Non-Hispanic White, and 12% uninsured. Out of our total patient pool, 7.6% experienced PDI, 13.3% experienced PHN, and 59.5% experienced some form of PHP.

**Table 1:** Patient Characteristics ADI: Area Deprivation Index, PDI: Postoperative Diabetes Insipidus, PHN: Postoperative Hyponatremia, PHP: Postoperative Hypopituitarism ^1^Number (%), Mean (Standard Deviation)

Factors	Overall (N = 210)^1^	PDI (N = 16)^1^	PHN (N = 28)^1^	PHP (N = 125)^1^
Sex				
Female	106 (50%)	11 (69%)	11 (39%)	65 (52%)
Male	104 (50%)	5 (31%)	17 (61%)	60 (48%)
Age	52.30 (15.63)	39.00 (19.42)	54.14 (16.83)	53.14 (15.18)
ADI	57.47 (25.32)	72.88 (26.31)	50.57 (30.62)	57.34 (25.54)
Race/Ethnicity				
Hispanic	24 (11%)	6 (38%)	3 (11%)	12 (9.6%)
Non-Hispanic African American	68 (32%)	3 (19%)	4 (14%)	41 (33%)
Non-Hispanic White	118 (56%)	7 (44%)	21 (75%)	72 (58%)
Insurance				
Private	128 (61%)	6 (38%)	18 (64%)	73 (58%)
Public	56 (27%)	6 (38%)	8 (29%)	36 (29%)
Uninsured	26 (12%)	4 (25%)	2 (7.1%)	16 (13%)
Tumor Volume	7.38 (18.80)	3.53 (3.82)	7.42 (12.37)	8.80 (21.97)

Postoperative diabetes insipidus

Logistic univariate and multivariate analyses for PDI are shown in Table 2. Univariate analysis demonstrated significance at the p<0.05 level for race/ethnicity, age, and ADI. Hispanic patients were significantly more likely to experience PDI than their racial/ethnic counterparts (p < 0.05). A higher (i.e., worse) ADI was significantly associated with increased odds of PDI (p < 0.05), as was younger age (p < 0.001). Taking into account all variables included in the study for multivariate analysis, younger age was associated with significantly increased odds of PDI (p < 0.05). There was no association between tumor volume and PDI incidence.

**Table 2:** Univariate and Multivariate Analysis of Postoperative Diabetes Insipidus OR: Odds Ratio, CI: Confidence Interval, ADI: Area Deprivation Index

Factors	Univariate		Multivariate	
	OR	95% CI	OR	95% CI
Sex				
Female	-	-	-	-
Male	0.44	0.13, 1.25	0.72	0.20, 2.37
Age	0.94	0.91, 0.98	0.96	0.92, 1.00
ADI	1.03	1.01, 1.06	1.02	1.00, 1.05
Race/Ethnicity				
Hispanic	-	-	-	-
Non-Hispanic African American	0.14	0.03, 0.58	0.32	0.04, 2.10
Non-Hispanic White	0.19	0.06, 0.65	0.32	0.06, 1.53
Insurance				
Private	-	-	-	-
Public	2.44	0.73, 8.15	3.00	0.77, 12.2
Uninsured	3.70	0.89, 14.0	1.20	0.21, 6.07
Tumor Volume	0.93	0.80, 1.01	0.95	0.81, 1.01

Postoperative endocrine status

The results of univariate and multivariate analyses are shown in Table 3. No factors of interest, including socioeconomic/demographic variables and preoperative tumor volume, were associated with increased odds of PHP.

**Table 3:** Univariate and Multivariate Analyses of Postoperative Endocrine Function OR: Odds Ratio, CI: Confidence Interval, ADI: Area Deprivation Index All analyses are conducted with postoperative hypopituitarism (PHP) as the primary factor of interest.

Factors	Univariate		Multivariate	
	OR	95% CI	OR	95% CI
Sex				
Female	-	-	-	-
Male	1.15	0.67, 1.97	1.39	0.79, 2.47
Age	0.99	0.97, 1.00	0.99	0.97, 1.01
ADI	1.00	0.99, 1.01	1.00	0.99, 1.01
Race/Ethnicity				
Hispanic	-	-	-	-
Non-Hispanic African American	0.64	0.26, 1.60	0.59	0.20, 1.75
Non-Hispanic White	0.70	0.30, 1.66	0.59	0.22, 1.62
Insurance				
Private	-	-	-	-
Public	0.66	0.34, 1.24	0.72	0.36, 1.41
Uninsured	0.76	0.31, 1.73	0.49	0.17, 1.29
Tumor Volume	0.98	0.95, 1.00	0.98	0.95, 1.00

Postoperative hyponatremia

No factors of interest correlated with PHN. The results of univariate and multivariate analyses are shown in Table 4.

**Table 4:** Univariate and Multivariate Analyses of Postoperative Hyponatremia OR: Odds Ratio, CI: Confidence Interval, ADI: Area Deprivation Index

Factors	Univariate		Multivariate	
	OR	95% CI	OR	95% CI
Sex				
Female	-	-	-	-
Male	1.69	0.76, 3.90	1.55	0.65, 3.77
Age	1.01	0.98, 1.04	1.01	0.98, 1.04
ADI	0.99	0.97, 1.00	0.99	0.98, 1.01
Race/Ethnicity				
Hispanic	-	-	-	-
Non-Hispanic African American	0.44	0.09, 2.37	0.24	0.04, 1.57
Non-Hispanic White	1.52	0.47, 6.83	0.87	0.21, 4.57
Insurance				
Private	-	-	-	-
Public	1.02	0.39, 2.44	0.95	0.33, 2.56
Uninsured	0.51	0.08, 1.93	0.45	0.06, 2.07
Tumor Volume	1.00	0.97, 1.02	1.00	0.97, 1.02

Exploratory analysis of race/ethnicity

As part of our data analysis, we sought to identify associations between race/ethnicity and socioeconomic/demographic factors of interest to better explain our other findings. The results of univariate and multivariate analyses, with Hispanic race/ethnicity serving as a comparison cohort to respective racial/ethnic counterparts, are displayed in Table 5 and Table 6, respectively. On both univariate and multivariate analyses, Hispanic patients were significantly more likely to be younger and uninsured compared to their respective racial/ethnic counterparts (p < 0.01). On univariate analysis, Hispanic patients were significantly more likely to live in areas with a higher (i.e., worse) ADI than Non-Hispanic White patients (p < 0.05).

**Table 5:** Univariate Analysis of Race/Ethnicity Against Other Factors of Interest OR: Odds Ratio, CI: Confidence Interval, ADI: Area Deprivation Index All comparisons are against the Hispanic racial/ethnic study cohort.

Factors	Non-Hispanic African American		Non-Hispanic White	
	OR	95% CI	OR	95% CI
Sex				
Female	-	-	-	-
Male	1.40	0.55, 3.58	1.45	0.60, 3.52
Age	1.10	1.06, 1.14	1.07	1.03, 1.10
ADI	1.00	0.98, 1.02	0.98	0.96, 1.00
Insurance				
Private	-	-	-	-
Public	1.10	0.26, 4.61	1.23	0.31, 4.91
Uninsured	0.04	0.01, 0.17	0.08	0.03, 0.25
Tumor Volume	1.00	0.98, 1.03	0.98	0.95, 1.01

**Table 6:** Multivariate Analysis of Race/Ethnicity Against Other Socioeconomic Factors OR: Odds Ratio, CI: Confidence Interval, ADI: Area Deprivation Index All comparisons are against the Hispanic racial/ethnic study cohort.

Factors	Non-Hispanic African American		Non-Hispanic White	
	OR	95% CI	OR	95% CI
Sex				
Female	-	-	-	-
Male	1.31	0.42, 4.13	1.19	0.41, 3.41
Age	1.09	1.05, 1.14	1.06	1.02, 1.10
ADI	1.01	0.99, 1.04	0.99	0.97, 1.01
Insurance				
Private	-	-	-	-
Public	0.63	0.13, 3.07	1.10	0.25, 4.94
Uninsured	0.05	0.01, 0.23	0.11	0.03, 0.35
Tumor Volume	1.00	0.98, 1.03	0.98	0.95, 1.01

## Discussion

This large retrospective cohort study of patients with pituitary adenoma who underwent transsphenoidal surgery revealed multiple socioeconomic correlates of adverse postoperative endocrine outcomes. To our knowledge, this study is the first of its kind to assess an array of postoperative endocrine complications with a focus on socioeconomic factors in pituitary adenoma patients.

The most striking findings of our study were the association of Hispanic ethnicity and ADI with increased incidence of PDI (Table 2). Taken together, these findings demonstrate that minority status and area of residence correlated with increased morbidity following pituitary adenoma resection.

Because minority status is not an inherent risk factor for PDI, we suspected that additional factors were at play that predisposed Hispanic patients in our sample to greater postoperative morbidity. This prompted us to determine whether there were any associations between race/ethnicity and the other independent variables in our study, which might suggest underlying disparities contributing to increased morbidity. Our exploratory analysis demonstrated that Hispanic patients were more likely to be uninsured and younger than their respective counterparts.

These additional findings, when confirmed by and integrated with prior literature, suggest that there are systemic disparities in access to healthcare services that specifically predisposed Hispanic patients in our study to adverse postoperative endocrine outcomes. For example, a study by Markowitz et al. (1991) noted that individuals between the ages of 18 and 24 who were Hispanic were less likely to have insurance coverage than similarly aged individuals from other respective backgrounds [29]. The most compelling suggestion, based on this confirmation of our findings, is that Hispanic patients in our sample were more prone to PDI due to inadequate insurance coverage or implicit bias on the part of providers, preventing earlier screening that resulted in larger tumors and increased disease burden.

The fact that preoperative tumor volume was not significantly associated with any other variables in our study, however, suggests that this might not be the complete picture. Our findings regarding tumor volume are confirmed by previous literature, which has not shown a correlation between larger tumors and increased incidence of PDI [30]. The correlations we identified on exploratory analysis may therefore provide alternative possible explanations for our findings. Lack of insurance coverage may correlate with food insecurity, lack of stable housing, and other socioeconomic stressors, decreasing patients’ ability to tolerate the physical stress of undergoing transsphenoidal surgery. This explanation is reinforced by the correlation in our data between worse ADI and increased incidence of PDI.

Another possibility is that Hispanic patients in our sample were more likely, because of their younger average age, to be aggressively treated by the operating neurosurgeons so as to prevent tumor recurrence, inadvertently resulting in more frequent PDI. Given that overall, patients with PDI were significantly younger on average than patients without PDI, this approach toward aggressive resection may have been universally utilized on all younger patients but was especially impactful on Hispanic patients in our sample due to economic and social disadvantages.

Although our study is the first of its kind to demonstrate a link between ethnicity and adverse postoperative endocrine outcomes, our additional findings confirmed by medical literature give us reason to suspect that this link is inherently tied to additional markers of socioeconomic status, in particular, insurance status and age. Together, these paint a broader picture where disparities in screening and increased socioeconomic burden predispose minority patients to increased postoperative endocrinopathy. Although further research will be beneficial for gaining additional insights into these findings, we believe that interventions aimed at addressing socioeconomic disparities among vulnerable patients, as well as increasing provider awareness of implicit bias, can serve as a useful starting point. These interventions will not only increase the likelihood of providers detecting symptomatic pituitary adenoma patients more quickly but also decrease the likelihood of patients being predisposed to greater postoperative burden when transsphenoidal resection is required.

This analysis was a retrospective chart review, which presents some limitations. Although we present statistically significant associations, we cannot demonstrate any causality and can only suggest potential underlying factors. Our study is also limited by the geographic location of our patients, most of whom live in North Carolina and all of whom received treatment at our institution.

Another limitation to our results is in the calculation of ADI using Neighborhood Atlas, whose latest version utilizes data from the American Community Survey ranging between 2014 and 2018. Given that some of our surgical records extend into 2020, it is possible that some of our patients were affected by recent shifts in regional or national socioeconomic factors that Neighborhood Atlas was unable to capture. For example, patients in our sample who received operations before May 2020 (the cutoff for our patient data) may have been significantly affected by the COVID-19 pandemic in ways that Neighborhood Atlas has not been updated to account for. However, because the majority of our patients’ operations took place before the onset of pandemic restrictions, we suspect that any influence of the pandemic on patients’ postoperative endocrine outcomes is minimal in our data set.

One of the most significant limitations of our study is that patient charts were read from our institution’s Electronic Medical Record (EMR), which records race and ethnicity as a combined field that is widely accessible. Although we have attempted to parse the two as sensitively and effectively as possible, with the categories our institution’s EMR provides in that combined field, we suspect that the nuance of patients’ subjective social status may not be adequately captured by their self-reported race/ethnicity, as they are capable of entering it on our institution’s EMR. Given our focus on select demographic cohorts, our findings cannot be extrapolated to individuals whose racial and ethnic identities fall beyond the scope of our study.

Although socioeconomic factors and demographics demonstrated associations with postoperative endocrine outcomes, this analysis cannot solely account for these findings. By exploring these associations further, via examining the associations between factors and demographics for additional insight, we have attempted to parse out possible explanations for our findings. However, we emphasize that this work should serve as a foundation for future studies, given the complicated interplay between socioeconomic status and health. While we have suggested preliminary preventative care measures to address the disparities we have identified, we welcome additional findings that lend themselves to advanced interventions targeted toward this patient population.

## Conclusions

We have demonstrated significant associations between multiple socioeconomic factors and postoperative endocrine outcomes in pituitary adenoma patients undergoing transsphenoidal surgery. In particular, worse ADI and Hispanic ethnicity are significantly associated with an increased incidence of PDI. This trend is inherently tied to other known factors in the literature such as age and insurance status. The associations we have identified, and the recommendations we have provided for initial interventions, may be useful to clinicians and surgeons managing pituitary adenoma patients, by providing additional resources and consideration for patients whose socioeconomic backgrounds may complicate their treatment course.
